# Psychosocial Stress Before a Nap Increases Sleep Latency and Decreases Early Slow-Wave Activity

**DOI:** 10.3389/fpsyg.2019.00020

**Published:** 2019-01-25

**Authors:** Sandra Ackermann, Maren Cordi, Roberto La Marca, Erich Seifritz, Björn Rasch

**Affiliations:** ^1^Division of Cognitive Biopsychology and Methods, Department of Psychology, University of Fribourg, Fribourg, Switzerland; ^2^Sleep & Health Zürich, University of Zurich, Zurich, Switzerland; ^3^Division of Clinical Psychology and Psychotherapy, Department of Psychology, University of Zurich, Zurich, Switzerland; ^4^Department of Psychiatry, Psychotherapy and Psychosomatics, Psychiatric Hospital, University of Zurich, Zurich, Switzerland

**Keywords:** sleep, stress, cortisol, cognition, emotion

## Abstract

Sleep disturbances are an important risk factor for stress-related diseases such as burnout or depression. In particular, slow-wave activity (SWA) during sleep might be eminently relevant for optimal maintenance of mental health and cognitive functioning. In spite of the clinical importance and the pertinence of stress-related processes in everyday life, the physiological mechanisms of the association between stress, sleep, and cognition are not well-understood. In the present study, we carefully mapped the time course of the influence of a psychosocial stressor on sleep architecture and sleep-related oscillations during a midday nap. We induced stress using a psychosocial laboratory stressor, the Montreal Imaging Stress Task, vs. a neutral control task. Afterward, participants were allowed to take a 90-min nap (*n* = 20) or stayed awake (*n* = 19) and cortisol was measured via saliva samples. We hypothesized that stress would decrease sleep efficiency and SWA in a time-dependent manner, with impairing effects on cognitive functioning. Psychosocial stress resulted in increased cortisol levels, which were elevated throughout the study interval. In the nap group, psychosocial stress increased sleep latency, but had only minor effects on sleep architecture. Still, SWA in the first 30 min of sleep was significantly reduced, whereas alpha activity was enhanced. These effects vanished after approximately 30 min. No impairing effect on cognitive functioning occurred. Our results show that psychosocial stress before sleep has an impact on sleep latency and early SWA during sleep. In contrast to our hypothesis, the effects were rather small and short-lasting. Importantly, cognitive functioning was maintained. We conclude that the effects of psychosocial stress before a nap are possibly better compensated than previously believed.

## Introduction

Sleep is critical for our mental health and well-being, and sleep disturbances are an important risk factor for stress-related syndromes such as burnout or depression (Hall et al., 2000; Söderström et al., 2004, 2012; Ekstedt et al., 2006, 2009; Sonnenschein et al., 2007; Armon et al., 2008; Willert et al., 2010).

In particular, slow-wave sleep (SWS) is important to maintain physical and mental health, and its characteristic slow-wave activity (SWA) has been shown to be functionally related to optimal recovery and brain plasticity (Finelli et al., 2001; Anderson and Horne, 2003; Tononi and Cirelli, 2006). In addition, SWS and SWA are critical for processes of sleep-associated memory consolidation and vigilance (Van Der Werf et al., 2009, 2011; Diekelmann and Born, 2010; Ackermann and Rasch, 2014).

Psychosocial stress has been reported to play a major role for the development and maintenance of sleep disturbances (Åkerstedt, 2006; Kim and Dimsdale, 2007). In previous studies, psychosocial stress and perseverative cognition [e.g., rumination (thoughts of past stressful events) and/or worry (feared events in the future)], that go along with psychosocial stress (Brosschot, 2010), have been associated with prolonged sleep onset latency, worse sleep efficiency, shorter as well as more fragmented sleep, more stage 1 sleep, less REM sleep, less SWS and prolonged SWS latency (Kecklund and Åkerstedt, 2004; Åkerstedt et al., 2007, 2014; Vandekerckhove et al., 2011; Wuyts et al., 2012). Moreover, in a sample of subjects with primary insomnia, higher stress levels were associated with decreased SWA during non-REM sleep (Hall et al., 2000).

As a physiological consequence stress alters hypothalamic–pituitary–adrenal (HPA) axis activity, and acute stress and stress induction lead to increased activation of the HPA axis, resulting in an increase in cortisol levels (Dickerson and Kemeny, 2004). Thus, effects of stress on sleep might be largely due to prolonged increases in cortisol due to the stressful experience. Interestingly, the reported effects of cortisol on sleep architecture are not completely consistent. On the one hand, four studies in humans focusing on acute stress and the association of HPA axis reactivity and sleep reported associations between subjective sleep measures and the cortisol response to a physiological (Goodin et al., 2012) or a psychosocial stressor (Räikkönen et al., 2010; Pesonen et al., 2012; Bassett et al., 2015). On the other hand, effects of cortisol administration on sleep show inconsistent results (Friess et al., 1995). In a study focusing on the effects of glucocorticoids on memory consolidation across sleep, mere direct infusion of a low dose of cortisol during early SWS-rich sleep did not change sleep architecture (Plihal and Born, 1999). Also administration of fludrocortisone before sleep or infusion of hydrocortisone during night sleep did not alter sleep architecture, except for a reduction of REM sleep after the infusion of hydrocortisone (Groch et al., 2013). It is thus questionable if cortisol release after stress induction can explain the effects of stress on sleep. Interestingly, sleep-associated memory consolidation was impaired in both above-mentioned studies, possibly due to a more fine-grained cortisol-related alteration on brain oscillations during sleep.

In the present study we focus on the effects of a psychosocial laboratory stressor on cortisol response and sleep measured with polysomnography. We were interested on the exact time course of the effect of acute stress on sleep architecture and sleep-related brain oscillations, in particular SWA. In addition, cortisol responses and cognitive functioning were measured. We hypothesize that a higher stress-level, going along with an increase in cortisol levels, is associated with worse sleep efficiency and lower SWA, leading to an impaired performance in cognitive tests.

## Materials and Methods

### Participants

Forty subjects took part in all sessions of the experiments. One subject in the wake group was excluded due to missing saliva samples. The remaining 39 participants were aged between 18 and 33 years (mean age 23.69 ± 3.76 years [standard deviation]). 20 subjects were in the nap group (10 women, 10 men) and 19 subjects were in the wake group (13 women, 6 men).

Participants were students or employees from the Zurich area and received 200 CHF for their participation. They did not take any medication (except hormonal contraceptives) and reported no neurological or mental illness. None of the participants had shift work or intercontinental flights within 6 weeks prior to participation in the study, had irregular night-day rhythms nor was habitually taking naps. Participants were asked to refrain from caffeine and alcohol on the days the experimental session took place. This study was carried out in accordance with the recommendations of the guidelines of the ethics committee of the University of Zurich with written informed consent from all subjects prior to participation. All subjects gave written informed consent in accordance with the Declaration of Helsinki. The protocol was approved by the ethics committee of the University of Zurich.

### Procedure

**FIGURE 1:**
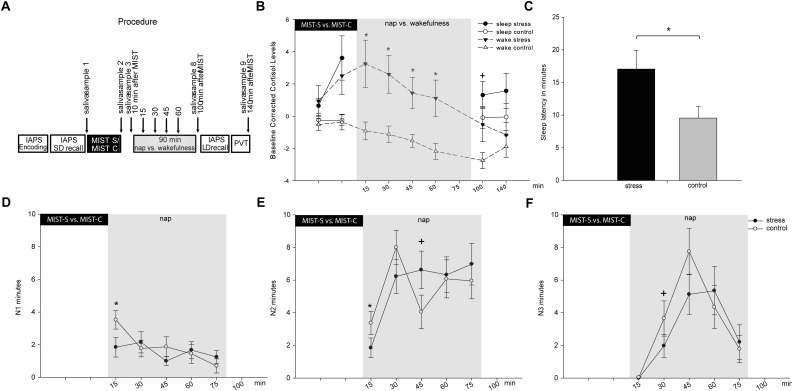
Displays the procedure of the experimental sessions and the main results on cortisol and sleep. **(A)** Procedure of the experiment. IAPS, picture memory task; SD, short delay; LD, long delay; PVT, psychomotor vigilance task. In **(B)** Baseline (sample 1) corrected cortisol values for wake and nap groups are reported for both conditions (stress in black versus control in white) separately. **(C)** Shows the overall effect of stress condition on sleep latency. **(D)** Shows effects in N1, **(E)** shows effects in N2 and **(F)** shows effects in N3 across the nap in 15 min segments. Asterisks indicate significant differences with *p* ≤ 0.05, trends are marked with +. The error bars represent standard errors of the marginal estimated means.

All subjects spent an adaption nap in the sleep laboratory 1 week before the first experimental session (sleep data see Table 1) and kept sleep diaries between each experimental session. Each subject took part in two experimental sessions (stress vs. control session) separated by 1 week, according to a balanced cross-over design (Figure 1). Each session started with application of electrodes, during which participants filled in questionnaires (see Materials and Methods). Afterward, participants performed one of two versions of the picture memory task, and short-delay free recall of the picture memory task was tested after 10 min (see Materials and Methods for an explanation of the tasks). During these 10 min, participants performed a working memory task (n-Back). Then either the laboratory stressor or the control condition was applied. 18 subjects started with the stress condition, 21 subjects started with the control condition. This task was followed by either a 90 min nap period (nap group) or a period of wakefulness (wake group). The wake group watched a documentary during the 90 min interval. Afterward, participants freely recalled the pictures again. Psychomotor vigilance was tested, and the session ended with filling in further questionnaires. Testing started always between 11:30 and 13:00 and ended between 17:00 and 19:00. Naps started between 14:10 and 16:05 and ended between 15:40 and 17:35.

### Psychosocial Stress Test

For stress induction we used the Montreal Imaging Stress Task (MIST) (Dedovic et al., 2005; Marca et al., 2011) as this task could be applied in the sleep laboratory while participants sat in front of a computer monitor. The MIST is a standardized computerized stress task combining challenging arithmetic problems and social-evaluative threat in the stress condition (MIST-S). The control condition (MIST-C) contains neither time pressure nor social evaluation.

Before the MIST-S started, the experimenter introduced an investigator impersonating a fictitious study leader who came by to check if everything was going well with the study. Then the experimenter explained the task to the subject and told her/him that data could only be used if she/he was performing well and fast. The difficulty of the task adapted to the performance of the subjects, in order to create a 45 to 50% performance range. A mock performance indicator showed the subjects that their task performance was poor compared to a control sample. One round had a duration of 4 min.

After the first round the experimenter entered the room and told the subject, that her/his performance was bad and asked the subject what the reason for the bad performance was (e.g., was there a problem?). The experimenter also told the study leader that it was an exception that the task did not go well and put pressure on the subject by telling her/him that it is very important that is concentrating on the task. After the second round, the experimenter called the study leader, who entered the room and put additional pressure on the participants (e.g., What is the problem?/Your performance was quite bad/Did you have math problems in the past?/It is important that you are doing well, the study costs a lot of energy and money for us). During the third round the study leader stayed in the room and repeatedly asked the subjects if she/he was stressed, looked for reasons why the subject was performing so bad and commented on what would have been the correct solution of the math problem. After the third round the study leader commented that the subjects performed bad again and left the room and told the experimenter to go on with the experiment.

In the control condition (MIST-C) the participants also had to solve math problems but they received neutral feedback after each round and were told, that their performance will not be analyzed. They were neither under time pressure nor did they receive any social evaluation nor was the study leader present.

Participants took part in the stress and the control condition in random order. If the participants performed on the stress condition in the first experimental session, they got following debriefing after the experimental session: “the next experimental session will be similar but with one big difference: there will be no more stress tasks and you will take part in the control condition. Thus, we are able to compare effects of stress vs. no stress on sleep. Do you have any questions?” After the second experimental session all subjects received a detailed debriefing about the MIST.

### Cortisol and Salivary Alpha-Amylase

Cortisol was measured via saliva samples using Salivette collection tubes (Sarstedt, Sevelen, Switzerland). In the nap group as well as in the wake group saliva samples (Figure 1A) were taken before the start of the MIST (sample 1, used as baseline sample), directly after the MIST (sample 2), 10 min after the MIST (sample 3), 100 min after the MIST (directly after 90 min of nap/wake; sample 8) and 140 min after the MIST (sample 9). In the wake group, we took four additional samples during the time the nap group took the nap: 15 min (sample 4), 30 min (sample 5), 45 min (sample 6), and 60 min (sample 7) after the third saliva sample. Cortisol and salivary alpha-amylase (sAA) values were baseline corrected with respect to sample 1 before the analysis (also see paragraph Stress Induction and Cortisol results section). After saliva collection salivettes were stored in the fridge and then frozen at -20°C. Cortisol and sAA levels were analyzed by the laboratory of the division of clinical psychology and psychotherapy of the University of Zurich. For cortisol and sAA analysis, saliva samples were centrifuged at 3000 rpm for 10 min after thawing. Concentrations of salivary free cortisol were measured using a commercially available enzyme immunoassay (IBL, Hamburg, Germany) with intra- and inter-assay precision of 3.2 and 4.2%, respectively.

Concentrations of sAA were measured using a commercially available assay (Alpha-Amylase EPS Sys. Roche Diagnostics).

### Sleep Data

#### Polysomnographic Recordings

The electroencephalogram (EEG), the electromyogram (EMG), and the electrocardiogram (ECG) were recorded in the wake as well as the nap group throughout the whole experimental session. We only analyzed data of the nap group during the nap. EEG was recorded using a high-density 128-channel Geodesic Sensor Net (Electrical Geodesics, Eugene, OR, United States) with a sampling rate of 500 Hz. The maximum for the electrical impedance was set at 50 kOhm. Electrodes were physically referenced to Cz and re-referenced to both mastoids during preprocessing. Data was preprocessed with Brain Vision Analyzer 2.0 (Brain Products, Gilching, Germany).

#### Sleep Scoring

In the nap group EEG data was visually scored between the markers lights-off and lights-on by two independent raters using standardized criteria following the manual published by the American Academy of Sleep Medicine (AASM) (Iber et al., 2007). Accordance rate between the raters was 92.69 ± 4.96% (mean ± SD) for the whole nap and 94.78 ± 3.05% for N2 epochs. Both did not differ between stress vs. control condition (both *p* > 0.20). For sleep stage analysis, data was referenced to the mastoids and filtered according to the recommendations in the AASM manual. For the total time in bed, every 30-s epoch was scored as NREM sleep stages 1, 2, 3, or REM sleep. Sleep onset was defined by the first period in stage 1 sleep which was followed by stage 2 sleep. SWS latency and REM sleep latency were determined with reference to sleep onset.

For a more fine-grained analysis we additionally segmented the nap into 15 min episodes starting from lights-off. This allowed investigating the time course of possible stress effects on sleep simultaneously to cortisol probes in the wake group.

#### Fast Fourier Transformation

**FIGURE 2:**
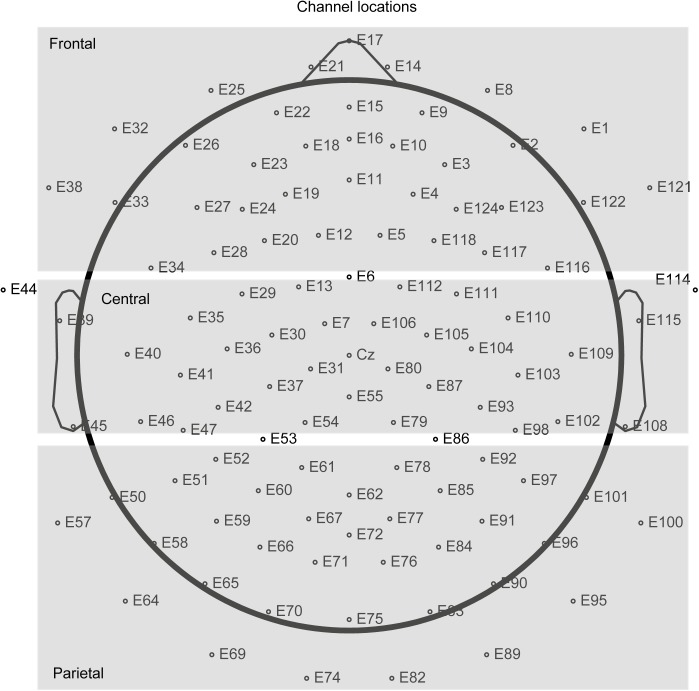
Displays how we defined the three regions frontal, central, parietal.

To investigate power differences across the whole nap period as well as within NREM sleep and the single 15-min segments, we subjected the data to spectral analysis using a fast Fourier transformation (FFT). Data was preprocessed by filtering between 0.1 and 35 Hz. The EEG signal was then segmented into equal sized episodes with 4096 data points (ca. 8 s) with 409 points overlap to compensate for later window-related data reductions. Artifact afflicted segments were deleted manually. The FFT was run with a 10% Hanning window and a resolution of 0.2 Hz. Power values for total power (0.5–50 Hz), SWA (0.5–4.5 Hz), theta (4.5–8 Hz), alpha (8–11 Hz), slow spindle (11–13 Hz), and fast spindle (13–15 Hz) were exported. This was done for the whole sleep episode, for only NREM sleep and for 15 min segments of the nap. Data was imported to SPSS. Based on topography (frontal, central, parietal), we created three regions by averaging electrode assemblies (see Figure 2 for more details). Topography “FCP” (frontal, central, parietal) was taken as within-subjects factor into the ANOVA.

### Heart Rate

Electrocardiogram was analyzed using Kubios HRV Version 3.1 (Tarvainen et al., 2014). Here, we used the automatic artifact correction on unfiltered data, eliminating ectopic beats and artifacts based on dRR series. We then segmented the data into 15 min episodes starting from lights off and corresponding to the sleep sections (see next paragraph). Within these segments, movement-related artifacts were eliminated in 5 min segments. Heart rate (HR) was then analyzed for each segment.

### Cognitive Tasks

#### Picture Memory Task

The picture memory task consisted of 90 pictures taken from the International Affective Picture System (IAPS) (Lang et al., 2008) as well as from the emo-pics set (Wessa et al., 2010) as well as from in-house standardized picture sets (some of the neutral pictures). Stimuli consisted of two sets (picture set 1 and picture set 2) of 30 positive, 30 negative, and 30 neutral pictures. In addition, four pictures showing neutral objects were presented to control for primacy and recency effects (two pictures were shown in the beginning of the presentation, the other two at the end). These pictures were not included in the analysis. Picture set 1 was presented in experimental session 1, picture set 2 was presented in experimental session 2. The two sets were counterbalanced for ratings of arousal [mean set 1: 4.66 ± 1.34 (SD), mean set 2 4.73 ± 1.31 (SD)] and valence [mean set 1: 4.87 ± 3.00 (SD), mean set 2 4.95 ± 2.15 (SD)] as well as for visual complexity and presence of humans.

The pictures were presented in a quasirandomized order so that a maximum of four pictures of the same category followed consecutively. A fixation-cross appeared for 500 ms before each picture. Then the picture was presented for 2.5 s. After presentation of each picture, subjects rated the presented picture according to its emotional valence [from 1 (very negative) to 5 (very positive)] and arousal [from 1 (low) to 5 (high)] to ensure deeper encoding of the pictures. Trials were separated by variable intertrial periods (9–12 s). Participants were told to memorize the pictures (intentional encoding).

For the free recall task, participants had to write down a short description of each picture. The participants were instructed to recall as many pictures as possible. The participants were given 20 to 25 min for this task. Participants were not told how many pictures they saw during picture presentation; therefore, no expectation of the number of pictures to be recalled was mentioned. Two independent and blind raters analyzed the recalled pictures and decided for each picture whether it could be recognized as one of the presented pictures. Afterward, a third independent and blind rater decided on pictures with diverging ratings.

Participants recalled the pictures 10 min after encoding (short-delay free recall) as well as approximately 120 min after encoding (long-delay free recall; after the nap or wake period). Memory retention over the nap or wake period was calculated as relative retrieval performance of picture set 1 with learning performance before the retention interval (short delay recall picture) set to 100% (long delay free recall/short delay free recall ^∗^ 100%).

#### Working Memory Task

Between picture presentation and recall, participants performed the 0- and 2-back versions of the n-Back working memory task (Gevins and Cutillo, 1993). Results of this task are not reported.

#### Psychomotor Vigilance Task

To assess vigilance, participants performed a psychomotor vigilance task (Dinges and Powell, 1985). One subject of the nap group had a missing value in this task due to technical problems.

### Questionnaires

For subjective sleep measures we used a subjective sleep quality questionnaire, the Schlaffragebogen A, revised version (SF-A/R) (Görtelmeyer, 2011) referring to the nap instead of the night. To measure circadian rhythm we used the German version of the Morningness-Eveningness Questionnaire (Horne et al., 1976). To check for depressive symptoms we used the Beck Depression Inventory (BDI) (Beck and Beamesderfer, 1974). To assess anxiety, we used the German Version of the State and Trait Anxiety Inventory (STAI) (Laux et al., 1981).

To assess the chronic daily stress levels we used the Trier Inventory for the Assessment of Chronic Stress (TICS) (Schulz and Schlotz, 1999).

To assess the influence of the stress or control task on positive and negative affect, subject filled in the German version of the Positive and Negative Affect Schedule (PANAS) (Breyer and Bluemke, 2016) in the beginning of the experimental session as well as before (missing in one subject of the wake group and in one subject of the nap group) and after the MIST (missing in one subject of the wake group). In addition, after the MIST, subjects rated (on a visual analog scale) how uncomfortable it felt to solve the math problems. This information is missing in three subjects of the wake group.

### Statistical Analysis and Data Reduction

We used SPSS (IBM SPSS Statistics Version 25) for data analysis. Unless indicated differently, values are presented as mean ± standard error of the mean (SEM).

Data was analyzed with mixed model repeated measures analysis of variance (ANOVA) and repeated measures ANOVAs. Significant main effects and interactions were further explored using uncorrected paired sample *t*-tests. To correct for multiple comparisons, we used the Fisher–Hayter procedure. Associations were explored with Pearson correlations and corrected for multiple testing according to the Bonferroni method. Chi-square tests were used to compare frequencies of traits between groups. *p* < 0.05 was considered significant.

## Results

### Questionnaires

#### Sleep Diaries Before Experimental Sessions and Circadian Rhythm

Subjective total sleep time did not differ between conditions, neither on the night before the experiment (7:28 vs. 7:10 h, for stress vs. control) nor during the entire week before the experiment (7:33 vs. 7:39 h, for stress vs. control, both *p* > 0.40). Similarly, subjective sleep quality did not differ between stress and control conditions, neither the night before the experiment (2.22 ± 1.20 vs. 2.44 ± 1.33) nor across the week before the experiment (2.50 ± 0.78 vs. 2.45 ± 1.01, both *p* > 0.60).

Morning and evening types, as measured with the D-MEQ, were equally distributed between the nap and the wake group (*p* = 0.996).

#### Mood and Anxiety

The wake and the nap group did neither differ in respect to the BDI (*p* = 0.862) nor the STAI state (measured before the adaption and the two experimental sessions, all *p* ≥ 0.30) nor the STAI trait (*p* = 0.522).

#### Subjective Stress Perception and Affect

Subjective stress levels increased after stress induction (question about MIST: “How uncomfortable did it feel to solve the math problems?”). Subjects in the nap as well as the wake group rated the stress condition as significantly more uncomfortable than the control condition [*F*(1,34) = 98.09, *p* < 0.001]. The interaction group ^∗^ condition as well as the main effect group did not reach significance (both *p* > 0.30).

In respect to positive and negative affect, as measured with the PANAS, subjects in the nap group as well as in the wake group did not differ in their scores before stress induction or the control condition (all *p* ≥ 0.217). After stress induction subjects scored higher on the negative affect scale in the stress condition than the control condition in the nap as well as the wake group (both *p* ≤ 0.003). In the wake group, in addition subjects scored lower on the positive affect scale after stress induction than after the control condition [*t*(17) = 2.40, *p* = 0.028]. The nap and the wake group did not differ in any of the measures (all *p* ≥ 0.10).

We also checked for differences in the chronic stress levels using the TICS. The subjects in the wake and the nap group didn’t differ in any of the subscales (all *p* ≥ 0.09).

#### Stress Induction and Cortisol

Because in the control condition, the nap and the wake group differed in cortisol levels before stress induction [sample 1; control condition: *F*(1,38) = 5.15, *p* = 0.029, stress condition: *F*(1,38) = 2.96, *p* = 0.093], we used sample 1 as baseline and used baseline-corrected cortisol values for all subsequent analyses. We show the time course of cortisol (baseline corrected samples 2 to 9) in Figure 1B.

We first conducted an analysis for the wake group including all eight time points of cortisol measurements (first measurement was used for baseline-correction, see Figure 1A for time points of cortisol measurements) including the factors condition and time. The interaction between stress and time reached significance [*F*(7,126) = 2.13, *p* = 0.045, $\eta_{p}^{2}$ = 0.106]. In addition, we found significant main effects of stress [*F*(1,18) = 8.76, *p* = 0.008, $\eta_{p}^{2}$ = 0.327] and time [*F*(1,126) = 9.15, *p* < 0.001, $\eta_{p}^{2}$ = 0.337]. Following up on the significant interaction, exploratory uncorrected *t*-tests showed a significant increase in baseline-corrected cortisol levels 10 min after the stress test as compared to the control condition [baseline-corrected sample 3, *t*(18) = 2.38, *p* = 0.028, see Figure 1B]. Cortisol responses remained elevated almost throughout the entire experimental period [baseline-corrected sample 4: *t*(18) = 2.74, *p* = 0.013, sample 5: *t*(18) = 3.13, *p* = 0.006, sample 6: *t*(18) = 3.14, *p* = 0.006, sample 7: *t*(18) = 2.80, *p* = 0.012], and were still marginally elevated in baseline-corrected sample 8 [*t*(18) = 1.97, *p* = 0.064] and became non-significant only 140 min after stress induction [baseline-corrected sample 9: *t*(18) = 0.699, *p* = 0.493], indicating a relatively long-lasting effect of stress induction on cortisol levels. Findings for baseline-corrected samples 4 to 7 also survived the control for multiple comparisons using the Fisher–Hayter Procedure (q_(0.05,15,120)_ = 4.898, Diff _crit._ = 2.72).

We then conducted the same analysis for the nap group. As no cortisol was collected during sleep, four time points were analyzed (samples 2 and 3 before the nap, samples 8 and 9 after the nap, see Figure 1A). We found a trend for an interaction between condition and time [*F*(3,57) = 2.33, *p* = 0.084, $\eta_{p}^{2}$ = 0.109]. In addition, the main effect condition reached significance [*F*(1,18 = 5.06, *p* = 0.037, $\eta_{p}^{2}$ = 0.210]. Following up on the interaction showed a significant difference between the stress and control condition for the increase in cortisol levels 10 min after stress induction [baseline-corrected sample 3, *t*(19) = 3.01, *p* = 0.007 exploratory uncorrected *t*-tests]. This finding also survived the control for multiple comparisons using the Fisher–Hayter Procedure (q_(0.05,7,48)_ = 4.351, Diff _crit._ = 2.65).

Descriptively, cortisol levels were still higher after the nap in the stress as compared to the control condition, although no significant differences occurred [baseline-corrected sample 8: *t*(19) = 1.36, *p* = 0.189; baseline-corrected sample 9: *t*(19) = 1.08, *p* = 0.293, exploratory uncorrected *t*-tests].

In a third step, we conducted an overall analysis across both groups (nap and sleep) and the within factors “condition” (stress vs. control) and “time” (baseline-corrected samples 2, 3, 8, and 9). Neither the three way interaction between condition, time and group nor the two way interaction condition and time reached significance [*F*(3,111) = 0.69, *p* = 0.559 and *F*(1,37) = 0.01, *p* = 0.932, respectively].

However, the interaction between time and group was significant [*F*(3,111) = 3.06, *p* = 0.031, $\eta_{p}^{2}$ = 0.076]. Following up on this interaction, cortisol levels were higher in sleep group as compared to the wake group after the 90 min period [baseline-corrected sample 8: *t*(38) = 2.21, *p* = 0.034, sample 9: *t*(38) = 2.45 *p* = 0.019, exploratory uncorrected *t*-tests]. These findings also survived the control for multiple comparisons using the Fisher–Hayter Procedure (q_(0.05,7,80)_ = 4.277, Diff _crit._ = 1.986). The nap and the wake group did not differ in respect to cortisol before the nap or wake period respectively (both *p* ≥ 0.708).

Also, the interaction between condition and time [*F*(3,111) = 3.06, *p* = 0.031, $\eta_{p}^{2}$ = 0.076] reached significance. Following up on this interaction, cortisol levels in the stress condition were higher than in the control condition after the MIST [baseline-corrected sample 2: *t*(38) = 2.10 *p* = 0.042, sample 3: *t*(38) = 3.867, *p* = 0.000] as well as directly after the 90-min period [baseline-corrected sample 8: *t*(38) = 2.395, *p* = 0.022, sample 9: *t*(38) = 1.301, *p* = 0.201, exploratory uncorrected *t*-tests]. These findings also survived the control for multiple comparisons using the Fisher–Hayter Procedure (q_(0.05,7,80)_ = 4.277, Diff _crit._ = 1.786). Cortisol in the stress and control condition did not differ 140 min after stress induction (*p* = 0.201).

In addition, the main effect condition [stress vs. control; *F*(1,37) = 9.56, *p* = 0.004, $\eta_{p}^{2}$ = 0.205], the main effect time [*F*(3,111) = 6.76, *p* < 0.001, $\eta_{p}^{2}$ = 0.155] and the main effect group [nap vs. wake; *F*(1,37) = 4.81, *p* = 0.035, $\eta_{p}^{2}$ = 0.115] reached significance.

To get one measure for the course of the cortisol levels over time (i.e., samples 1, 2, 3, 8, and 9) we additionally computed the area under the curve with respect to increase (AUCi) using the formula suggested by Pruessner et al. (2003). The interaction between the nap vs. wake group and stress vs. control did not reach significance (*p* > 0.90). As expected, in both groups the AUCi was larger in the stress condition as compared to the control condition [*F*(1,37) = 11.00, *p* = 0.002, $\eta_{p}^{2}$ = 0.229]. The main effect group (nap vs. wake) reached a trend level [*F*(1,37) = 3.79, *p* = 0.059, $\eta_{p}^{2}$ = 0.093], with a generally larger AUCi in the nap group than the wake group.

#### Stress Induction and Cortisol, Including Sex as Factor

We conducted an additional analysis adding sex as a between-factor to the model, as this factor may influence cortisol levels. The results are comparable to the ANOVA not including sex (see previous paragraph).

The three way interaction between the factors “condition,” “time,” and “group” did not reach significance [*F*(3,105) = 1.08, *p* = 0.361], neither did the two way interaction between condition and group [*F*(1,35) = 0.012; *p* = 0.913].

In contrast to the analyses not controlling for influences of sex, the two way interaction between condition and time only reached a trend [*F*(3,105) = 2.36, *p* = 0.075, $\eta_{p}^{2}$ = 0.063].

As in the previous analysis, the two way interaction between time and group [*F*(3,105) = 3.74, *p* = 0.026, $\eta_{p}^{2}$ = 0.097] as well as the main effect condition [*F*(1,35) = 8.22, *p* = 0.007, $\eta_{p}^{2}$ = 0.190], the main effect time [*F*(3,105) = 6.71, *p* > 0.005, $\eta_{p}^{2}$ = 0.161], and the main effect group [nap vs. wake; *F*(1,35) = 6.19, *p* = 0.018, $\eta_{p}^{2}$ = 0.150] reached significance.

In respect to sex, none of the interactions reached significance (trend for an interaction between group and sex: *F*(1,35) = 2.97, *p* = 0.094; all other *p* ≥ 0.203). Neither did the main effect of sex reach significance [*F*(1,35) = 0.03, *p* = 0.858].

#### Stress Induction and Salivary Alpha-Amylase

As an overall analysis we conducted a mixed model repeated measures ANOVA with the within factors “condition” (stress vs. control) and “time” (baseline-corrected samples 2, 3, 8, and 9) and the between subjects factor “group” (nap vs. wake). The three way interaction between condition, time and group did not reach significance [*F*(3,111) = 1.55, *p* = 0.215].

Neither did any of the two way interactions (all *F* ≤ 1.50, all *p* ≥ 0.22) nor any of the main effects reach significance (all *F* ≤ 1.38, all *p* ≥ 0.25).

### Sleep Parameters

When focusing on the total nap time, we found condition-related differences in sleep latency (see Table 2). After stress induction, subjects had a significantly longer sleep latency (17.05 ± 2.87 min) as compared to sleep latency after the control condition (9.03 ± 1.50 min), *t*(19) = 3.20, *p* = 0.005, see Figure 1C. This result also survived Bonferroni-correction for multiple comparisons (corrected significance threshold *p* = 0.006). However, we did not find any differences in any other sleep parameter (all *p* > 0.30). We also did not find differences in subjective sleep latency between the stress condition and the control condition [*t*(18) = 1.18, *p* = 0.255].

**Table 1:** Sleep parameters in the adaptation nap.

Sleep parameters	Baseline
Sleep length (min)	77.98 ± 2.18
Sleep efficiency	76.88 ± 3.82
%Wake	12.34 ± 3.06
%S1	11.20 ± 1.91
%S2	41.10 ± 2.88
%SWS	29.58 ± 4.94
%REM	5.77 ± 1.78
Sleep latency (min)	11.55 ± 1.98
Wake (min)	9.00 ± 2.18
S1 (min)	8.63 ± 1.54
S2 (min)	32.05 ± 2.33
SWS (min)	23.53 ± 4.06
REM (min)	4.78 ± 1.49


**Table 2:** Stress effects on sleep in total nap time.

Sleep parameters	Stress	Control	*p*
Sleep length (min)	66.35 ± 4.16	72.50 ± 4.45	0.320
Sleep efficiency	71.41 ± 4.44	75.48 ± 5.06	0.508
Sleep latency (min)	17.05 ± 2.87	9.03 ± 1.50	0.005^∗^
Wake (min)	2.63 ± 0.67	3.90 ± 1.45	0.427
S1 (min)	10.83 ± 1.87	10.48 ± 1.43	0.822
S2 (min)	32.13 ± 3.07	35.10 ± 2.80	0.531
SWS (min)	16.28 ± 3.13	17.98 ± 3.51	0.591
REM (min)	4.33 ± 1.45	4.88 ± 1.37	0.772


*Displays sleep parameters for the whole duration of the nap for the stress versus the control condition. *p*-values indicate the within group-comparison between those two conditions.*

Because we were interested in the time course of the influence of stress induction on sleep parameters, we conducted a more detailed analysis of sleep progression. Therefore, we segmented sleep into 15-min epochs starting after lights-off. We found a trend for an interaction between condition and time in N1 [*F*(5,95) = 2.07, *p* = 0.076], a significant interaction of condition and time in N2 [*F*(5,95) = 2.58, *p* = 0.031, $\eta_{p}^{2}$ = 0.119] and a trend for an interaction of condition and time in N3 [*F*(5,95) = 2.19, *p* = 0.062].

The most pronounced stress-related differences in sleep parameters appeared in the first 15 min after lights-off, with lower amounts of N1 and N2 sleep in the stress condition as compared to the control condition (see Figures 1D,E and Table 3). Those marked differences diminished in minute 15–30 and completely abolished across the following 15-min episodes. In addition, a trend for reduced SWS after stress occurred after 15–30 min and after 30–45 min (see Figure 1F). No other effects were significant, except an increase in N2 sleep in the control group in the last 15-min segment of the nap (not displayed in the Figure, but see Table 3). However, none of the *post hoc* comparisons remained significant when correcting for multiple comparisons using the Fisher–Hayter correction.

**Table 3:** Sleep stage differences in 15 min segments.

	Stress	Control	*p*
0–15 min	Mean ± SEM	Mean ± SEM	
Wake minutes	0.15 ± 0.13	0.30 ± 0.22	0.57
N1 minutes	1.85 ± 0.60	3.53 ± 0.57	0.026^*^
N2 minutes	1.85 ± 0.60	3.38 ± 0.71	0.053^*^
N3 minutes	0.00 ± 0.00	0.05 ± 0.03	0.163
REM minutes	0.00 ± 0.00	0.00 ± 0.00	N/A
15–30 min			
Wake minutes	0.35 ± 0.33	0.73 ± 0.39	0.49
N1 minutes	2.15 ± 0.65	1.78 ± 0.51	0.61
N2 minutes	6.23 ± 1.05	8.00 ± 1.06	0.26
N3 minutes	1.98 ± 0.71	3.65 ± 1.08	0.07^+^
REM minutes	0.00 ± 0.00	0.00 ± 0.00	N/A
30–45 min			
Wake minutes	0.63 ± 0.37	1.28 ± 0.49	0.25
N1 minutes	1.00 ± 0.27	1.88 ± 0.62	0.188
N2 minutes	6.63 ± 1.15	4.05 ± 1.03	0.074^+^
N3 minutes	5.13 ± 1.24	7.75 ± 1.41	0.092
REM minutes	0.00 ± 0.00	0.00 ± 0.00	N/A
45–60 min			
Wake minutes	1.10 ± 0.36	2.98 ± 1.09	0.101
N1 minutes	1.68 ± 0.51	1.45 ± 0.58	0.724
N2 minutes	6.33 ± 1.10	6.08 ± 1.16	0.887
N3 minutes	5.35 ± 1.48	4.35 ± 1.31	0.512
REM minutes	0.50 ± 0.35	0.35 ± 0.35	0.343
60–75 min			
Wake minutes	2.65 ± 1.07	3.58 ± 1.35	0.559
N1 minutes	1.25 ± 0.40	0.70 ± 0.43	0.374
N2 minutes	6.98 ± 1.28	5.95 ± 1.10	0.577
N3 minutes	2.20 ± 1.06	1.78 ± 0.84	0.696
REM minutes	1.88 ± 0.83	2.95 ± 1.05	0.441
75–90 min			
Wake minutes	4.10 ± 1.37	3.35 ± 1.30	0.70
N1 minutes	2.95 ± 0.75	2.10 ± 0.82	0.211
N2 minutes	3.73 ± 0.77	7.25 ± 1.07	0.017^*^
N3 minutes	1.53 ± 0.73	0.40 ± 0.23	0.160
REM minutes	2.30 ± 0.93	1.50 ± 0.54	0.417


*Displays sleep parameters in the six 15 min segments (15 min after lights-off, minutes 15–30, 30–45, 45–60, 60–75, and 75–90) for the stress versus the control condition. *p*-values indicate the withingroup-comparison between those two conditions. Reported results are explorative uncorrected *t*-tests. Asterisks indicate significant differences with *p* ≤ 0.05, trends are marked with +. No comparison remained significant when correcting for multiple comparisons using the Fisher–Hayter correction (q_(*0.05,11,80*)_ = 4.686, Diff_*crit*_ = 4.537).*

### Frequency Analysis

We calculated repeated measure ANOVAs with the within-subject factors “condition” (stress vs. control) and “FCP” (frontal vs. central vs. parietal topography). Neither for the entire nap period (*p* = 0.45) nor the analyses based on NREM sleep episodes (*p* > 0.90), did total power differ depending on stress condition or control condition. We still corrected for possible general and unspecific power differences between the sessions by reporting relative values for which we set the amount of total power (0.5–50 Hz) to 100%. The reported values are thus percentage of power in the respective bands relative to total power during the session.

#### Frequency Analysis for the Whole Nap Period and NREM Sleep

We analyzed and compared differences in power in frequency bands of SWA (0.5–4.5 Hz), theta (4.5–8 Hz), alpha (8–11 Hz), slow (11–13 Hz), and fast spindles (13–15 Hz). No significant main effects or interactions with stress appeared in any of the frequency bands, neither in the entire nap period (all *p* > 0.07) nor NREM sleep episodes (all *p* > 0.15).

#### Frequency Analysis for the Course of Sleep, Measured by 15 min Segments

We analyzed the power differences between conditions in the 15 min nap segments in repeated measure ANOVAs with the within-subject factors “time” (five segments, excluding the last 90 min in which data of one subject was missing), “condition” (stress vs. control) and “FCP” (frontal vs. central vs. parietal topography). We analyzed the relative power in the frequency bands by setting total power (0.5–50 Hz) of each segment to 100%. This took account for potential overall power differences between the two sessions. Thus, reported values are percentage of power relative to total power in that segment.

**FIGURE 3:**
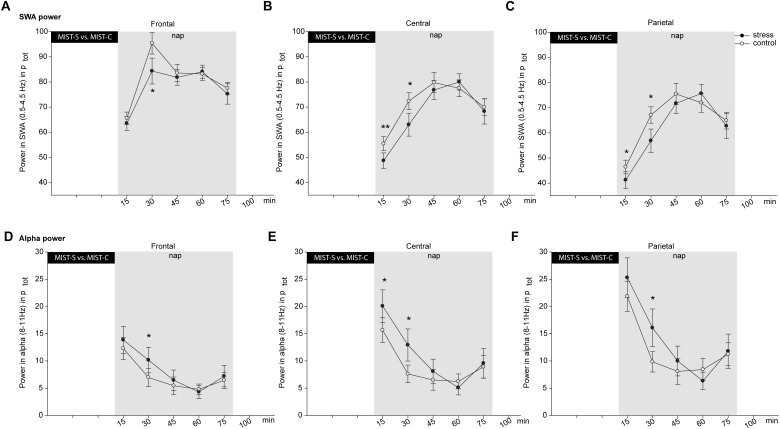
Effects of stress (black dots) versus control (white dots) on relative SWA **(A–C)** and alpha power **(D–F)** separately for frontal **(A,D)**, central **(B,E)** and parietal **(C,F)** regions. Asterisks indicate significant stress effects in a within-subjects ANOVA with *p* ≤ 0.05. All indicated significant *post hoc* comparisons survived the correction for multiple comparisons using the Fisher–Hayter Procedure. The error bars represent standard errors of the marginal estimated means.

For power in the SWA frequency band, a significant three-way interaction between all three factors appeared [*F*(8,152) = 2.39, *p* = 0.019, $\eta_{p}^{2}$ = 0.11]. Also, the time ^∗^ condition interaction was significant [*F*(4,76) = 2.73, *p* = 0.035, $\eta_{p}^{2}$ = 0.13]. Following up on this interaction showed that SWA power after stress is lower (51.16 ± 2.93%) than after control (55.82 ± 2.57%) in the first 15 min [*t*(19) = -2.67, *p* = 0.015] and the second segment [*t*(19) = -2.39, *p* = 0.027, 68.08 ± 4.66 vs. 78.28 ± 3.48%], but not in later segments (all *p* > 0.35, exploratory uncorrected *t*-tests). Only the difference in the second segment survived the control for multiple comparisons using the Fisher–Hayter Procedure (q_(0.05,9,60)_ = 4.55, Diff _crit._ = 8.89). For the three-way interaction, differences in the first and second time segment were observed in central and parietal recording sites, in frontal electrodes at the first time point, corrected for multiple comparisons using the Fisher–Hayter Procedure (see Figures 3A–C).

For the theta band, the three-way interaction between the three factors was only a statistical trend [*F*(8,152) = 1.77, *p* = 0.088, $\eta_{p}^{2}$ = 0.09]. The other effects with condition were *p* > 0.20.

Also, for alpha power we found a significant three-way interaction [*F*(8,152) = 3.25, *p* = 0.002, $\eta_{p}^{2}$ = 0.15, *q* = 5.5, Diff_crit._ = 2.18] and a significant time by condition interaction [*F*(4,76) = 2.51, *p* = 0.049, $\eta_{p}^{2}$ = 0.12]. Follow-up analyses showed that alpha power was higher after stress (19.74 ± 2.91 and 13.05 ± 2.83%) than control (16.59 ± 2.31 and 8.13 ± 1.68%) in the first [*t*(19) = 2.23, *p* = 0.038] and second segment [*t*(19) = 2.33, *p* = 0.031], but not later (all *p* > 0.30, exploratory uncorrected *t*-tests). No difference survived the control for multiple comparisons using the Fisher–Hayter Procedure (q_(0.05,9,60)_ = 4.55, Diff _crit._ = 6.67). For the three-way interaction, corrected significant *post hoc* differences were observed in central and parietal sites at the first and second time point, and in frontal sites at the second time point (see Figures 3D–F).

For slow spindle power, only the three-way interaction was significant [*F*(8,152) = 3.63, *p* = 0.001, $\eta_{p}^{2}$ = 0.16], while all others were *p* > 0.10. Follow-up analyses however revealed no condition-related differences in frontal, central, nor parietal electrodes (all *p* > 0.10).

No effects appeared in fast spindle power (all *p* > 0.20).

### Cortisol and Sleep

We correlated differences of cortisol increases after the stress vs. control condition of the MIST (i.e., baseline-corrected sample 3, stress minus control condition) with differences in sleep measures over the whole duration of the nap in the stress versus the control condition. Cortisol levels were not associated with any sleep measures (all p_uncorrected_ ≥ 0.27).

As we found time dependent effects in sleep parameters when comparing the stress condition and the control condition, we also conducted the same cortisol and sleep parameter correlations per 15 min of sleep (Bonferroni-corrected for multiple comparisons, corrected significance threshold *p* = 0.0016). We observed one negative correlation that survived correction for multiple testing: higher cortisol increase after stress induction vs. control significantly predicted a stronger reduction in N1 sleep in the stress vs. the control condition between 45 and 60 min (*r* = -0.70, p_uncorrected_ = 0.001). No further correlations did survive Bonferroni correction for multiple comparisons. Following correlations were significant on a nominal level: a negative correlation between cortisol increase after stress induction vs. control and N1 sleep in the stress vs. the control condition between 30 and 45 min (*r* = -0.486, p_uncorrected_ = 0.030) and a positive correlation cortisol between increase after stress induction vs. control and N1 sleep in the stress vs. the control condition between 60 and 75 min (*r* = 0.526, p_uncorrected_ = 0.017).

In addition, we correlated sleep parameters over the whole duration of the nap (stress – control condition) with cortisol levels after the nap (i.e., baseline-corrected samples 8 and 9, stress minus control condition), as sleep may influence cortisol as well. Sleep measures were not associated with the cortisol levels after the nap (all p_uncorrected_ ≥ 0.079).

Also, here we conducted cortisol and sleep parameter correlations per 15 min of sleep (Bonferroni-corrected for multiple comparisons, corrected significance threshold *p* = 0.00083).

Following correlations were significant on a nominal level: a positive correlation between N1 sleep in the first 15 min and cortisol after the nap (baseline-corrected sample 8) in the stress vs. control condition (*r* = 0.479, p_uncorrected_ = 0.033), negative correlations between N1 sleep between 15 and 30 min and cortisol levels after the nap (with baseline-corrected sample 8: *r* = -0.465, p_uncorrected_ = 0.039, with baseline-corrected sample 9: *r* = -0.528, p_uncorrected_ = 0.017).

### Heart Rate and Sleep

We analyzed heart rate during the nap and also segmented it into the same 15 min segments. Artifacts were rejected in 5-min epochs. In the ANOVA with stress condition by time, the main effect of time [*F*(4,72) = 4.23, *p* = 0.004, $\eta_{p}^{2}$ = 0.19], of stress condition [*F*(1,18) = 6.94, *p* = 0.017, $\eta_{p}^{2}$ = 0.28] and their interaction were significant [*F*(4,72) = 2.99, *p* = 0.024, $\eta_{p}^{2}$ = 0.14]. Follow-up exploratory uncorrected *t*-tests showed that for 15, 30, and 45 min, heart rate was faster in the stress than control condition [59.50 ± 1.90 vs. 55.55 ± 1.66, *t*(18) = 3.81, *p* = 0.001; 57.23 ± 1.72 vs. 54.08 ± 1.52, *t*(18) = 3.38, *p* = 0.003 and 57.15 ± 1.52 vs. 53.88 ± 1.54, *t*(18) = 3.43, *p* = 0.003]. All time points remained significant when correcting for multiple comparisons using the Fisher–Hayter Procedure (q_(0.05,9.60)_ = 4.55, Diff _crit._ = 2.55). Later time windows did not differ depending on stress condition (all *p* ≥ 0.25).

The difference in heart rate between stress vs. control did not correlate with difference in alpha power between stress vs. control (all p_uncorrected_ > 0.15). With SWA power difference, only in the episode of 45 min sleep the correlation was significant, indicating that a higher difference in heart rate between the conditions is associated with a lower SWA power difference. Correcting these analyses for multiple comparisons would however nullify it. All other correlations were p_uncorrected_ > 0.20.

### Subjective Stress Levels and Sleep

We analyzed whether acute (question about the MIST) and chronic [screening subscale of chronic stress (SSCS) of the TICS] subjective stress levels are associated with the sleep parameters as well as the memory parameters (per valence) in the stress versus the control condition. None of the correlations between the subjective stress parameters and the sleep parameters reached significance (all p_uncorrected_ ≥ 0.154). In respect to the memory parameters, we found a significant negative correlation between the acute stress level (stress minus control) and recall of negative pictures (stress minus control; *r* = -0.523, p_uncorrected_ = 0.018) in the nap group and a significant positive correlation between the acute stress level (stress minus control) and recall of positive pictures (stress minus control; *r* = 0.646, p_uncorrected_ = 0.007). However, these associations did not survive correction for multiple testing (Bonferroni corrected significance threshold *p* = 0.002).

### Cognitive Measures

#### Picture Memory Performance

We did not find a significant three-way interaction of condition (stress/control), group (nap/wakefulness), and valence (positive, negative, neutral) on memory consolidation (*p* = 0.718). Neither did any of the two-way interactions or main effects reach significance (all *p* ≥ 0.12).

In addition, we correlated recall of pictures (stress condition minus control condition) per valence (positive, negative, neutral) with sleep parameters (stress condition minus control condition). We found a nominally significant positive correlation between minutes non-REM sleep and recall of neutral pictures (*r* = 0.483, p_uncorrected_ ≥ 0.031). However, this correlation did not survive correction for multiple testing (Bonferroni corrected significance threshold *p* = 0.0006).

#### Psychomotor Vigilance Task

There was neither an effect of condition nor group nor the interaction of both factors on average reaction time measured in the PVT (all *p* > 0.20) or on error rate (all *p* > 0.20).

## Discussion

In the present study, we investigated, whether stress induction using a psychosocial stressor is associated with worse sleep quality during a nap and higher cortisol levels and whether these effects are time-dependent. Stress induction lead to a significant increase in cortisol levels which was present almost throughout the entire experiment. In the wake group, we detected stress-induced cortisol elevations throughout the wakefulness period until 100 min after stress-induction. However, in the nap group, changes in sleep-related EEG activity were only detectable until 45 min after stress-induction. These results may indicate that stress effects on sleep oscillations are not as long lasting as stress-induced cortisol elevations as measured in the wake group. One possibility is that the stress-induced differences in SWA and alpha activity vanish in spite of increased cortisol levels during sleep, suggesting that similar sleep depth can be achieved after stress while cortisol levels are still increased. An alternative explanation is that the increases in cortisol after stress induction vanish quicker as compared to wakefulness, so that cortisol and EEG changes during sleep occur in parallel. Future studies measuring cortisol also during the sleep period are needed to answer these questions.

In the present study we observed higher cortisol levels after the nap as compared to a period of wakefulness. This difference was statistically significant in the control group, whereas in the stress condition we saw this effect only on a descriptive level. Increase in cortisol values after a period filled with sleep might be possibly due to a cortisol awakening response (CAR) (Fries et al., 2009; Clow et al., 2010). Previous studies have shown that the CAR is larger after night time sleep as compared to 90 min naps (Devine and Wolf, 2016). These authors also showed associations between sleep stages and CAR after night time sleep (stage 2) as well as after a morning nap (stage 1), while there were no associations with CAR after afternoon naps. Interestingly, no CAR was observed after a nap duration of 60 or 50 min (Federenko et al., 2004; Devine and Wolf, 2016). According to these results, our nap duration might have been rather short to elicit a strong CAR (mean TST of 66.35 ± 4.16 min in the stress condition and 72.50 ± 4.45 min in the control condition). Furthermore, in the present study we report a negative association between cortisol after stress induction and N1 sleep toward the end of the nap.

Results indicate influences of psychosocial stress on sleep quality in a nap, in particular on sleep onset latency and sleep shortly after sleep onset. We found several time-dependent effects of stress induction on sleep. After the control condition, subjects show more N1 sleep and N2 sleep in the first 15 min of the nap than after the stress condition. In the last 15 min of the nap, subjects again have more N2 sleep after the control condition than after the stress condition. In addition, a trend for reduced SWS after stress occurred after 15–30 and 30–45 min. Sleep frequency analyses show that power in the SWA frequency band is lower and the alpha frequency band is higher after the stress condition than after the control condition in the first and second 15 min of nap. These results show a time-dependency of the effects of an acute psychosocial stressor on subsequent nap. To our knowledge, this is the first study showing time-dependent effects of psychosocial stress-induced cortisol changes on sleep. However, the effects are rather small and short-lasting.

Our results show parallels to previous studies on stress effects on sleep. Several studies focusing on psychosocial stress or pre-sleep arousal and its impact on sleep showed increased sleep onset latency and more stage 1 sleep (Wuyts et al., 2012; Åkerstedt et al., 2014).

Moreover, we found effects on SWA, which has also been associated with stress in a sample of patients with primary insomnia (Hall et al., 2000). SWA is crucial for optimal recovery, brain plasticity (Finelli et al., 2001; Anderson and Horne, 2003; Tononi and Cirelli, 2006) and sleep-associated memory consolidation and vigilance (Van Der Werf et al., 2009, 2011).

In the present study we were also interested, whether stress induction affects cognition in the nap and the wake group differently. However, in contrast to our hypothesis, we did not find any interactions of stress and sleep on a declarative memory task or on vigilance. In our study, effects of a psychosocial stressor on memory recall are not modulated by sleep nor does it impact on memory performance or vigilance. Even though in the present study sleep parameters change to some extend with elevated cortisol levels, this does not affect memory consolidation.

These findings stand in contrast to a previous study, showing that basal pre-learning cortisol levels influence memory consolidation across night sleep but not across the same period of wakefulness (Bennion et al., 2015). However, this study uses a different memory paradigm (scenes including a neutral or negative object) and recognition memory while in the present study we focus on free recall. Moreover, the effects of stress induction, as was done in the present study, may differ from effects of basal cortisol on memory. In an evening nap study, post-learning infusion of cortisol during sleep or wakefulness neither affected memory retention, which was tested after cortisol levels had returned to normal values. Cortisol had effects on the recall of temporal order; it was positively influenced in the wake group and negatively in the nap group (Wilhelm et al., 2011). However, the design as well as the memory task in our study differed from above mentioned studies. In addition, in the present study, cortisol was still elevated at time of recall which may also have influenced memory recall (also see de Quervain et al., 2009; Wolf, 2009 for reviews of cortisol effects on memory). Our results may also point to a different process when focusing on a psychosocial stressor as compared to basal cortisol or pharmacologically elevated cortisol during sleep. To answer these questions, studies using the same design and memory tasks for investigating effects of basal cortisol or cortisol elevation (through stress induction or pharmacologically induced) on memory consolidation and sleep are needed.

Moreover, the effect of stress on memory consolidation may be driven by cortisol and may be more pronounced in cortisol responders than non-responders (Stock and Merz, 2018), therefore this possibility should be analyzed in a larger sample.

In spite of the clinical importance and the pertinence of stress-related learning processes in everyday life, the behavioral, physiological as well as molecular mechanisms of the association between stress, sleep and memory are not well-known and merit more research.

As both the nap and the wake period took place at the same circadian time (midday – afternoon), we did not expect circadian differences in cortisol levels between the two groups. However, sleep architecture, time of day as well as nap duration may have a specific effect on cortisol levels, in particular on cortisol after waking up (Devine and Wolf, 2016). Future studies need to measure cortisol also during sleep to get more information on the course of cortisol during sleep.

In addition, it is not clear whether we can generalize the effects reported here to nighttime sleep. The effects of a stressor on a nap may differ from effects on night sleep due to circadian rhythmicity of cortisol. During the day, cortisol levels are higher, gradually decreasing throughout the day with lowest levels during the first half of the night (Fries et al., 2009; Clow et al., 2010). Moreover, although sleep architecture in naps generally follows the same pattern as sleep during the night (Maron et al., 1964), time of day may also influence sleep architecture (Karacan et al., 1970).

A further limitation of the study are confounding factors that may determine cortisol changes. All of our female participants used hormonal contraceptives due to practical reasons. In a large study investigating effects of basal cortisol on memory, results did not differ between naturally cycling women and women taking oral contraceptives (Ackermann et al., 2013). However, as compared to naturally cycling women, hormonal contraceptives have been shown to attenuate cortisol effects on memory (Kuhlmann and Wolf, 2005) and blunt the cortisol response to stress induction (Kirschbaum et al., 1999; Nielsen et al., 2013). Therefore, the effects of stress induction and sleep on memory might have been larger in naturally cycling women.

We did not include any women not taking hormonal contraceptives due to the larger variability it would have added to the sample and due to the fact, that the different menstrual phases may influence cortisol levels and response to the stressor (Kirschbaum et al., 1999; Maki et al., 2015).

A laboratory stressor probably has different effects on sleep than an everyday stressor, due to various differences such as duration. However, using a laboratory stressor allows for standardization of stress induction. Looking at everyday stressors, it cannot be concluded whether effects of stress lead to sleep impairments or whether effects of sleep impairments are a cause for increased stress perception. It is most likely a bidirectional relationship (Van Laethem et al., 2015).

In sum, stress induction using a psychosocial stressor only affects sleep stages and power spectra in the first 15 to 30 min of the nap. Cortisol levels normalize later across sleep to the level of the control condition. Therefore, we conclude that effects of a psychosocial stressor on sleep are time-dependent. Moreover, changes in sleep stages and power spectra are paralleled by changes in cortisol levels induced by stress. We conclude that the effects of psychosocial stress before a nap are possibly better compensated than previously believed.

## Author Contributions

SA and BR developed the study design. SA and RLM prepared the study and collected the data. SA and MC analyzed the data. All authors wrote the manuscript.

## Conflict of Interest Statement

The authors declare that the research was conducted in the absence of any commercial or financial relationships that could be construed as a potential conflict of interest.
